# An optimized whole blood assay measuring expression and activity of NLRP3-, NLRC4 and AIM2-inflammasomes

**DOI:** 10.1186/1546-0096-13-S1-O51

**Published:** 2015-09-28

**Authors:** L Grinstein, H Luksch, AAB Robertson, MA Cooper, S Winkler, A Rösen-Wolff

**Affiliations:** 1 University Hospital Carl Gustav Carus, Department of Pediatrics, Dresden, Germany; 2 University Hospital Carl Gustav Carus, Department of Pediatrics, Dresden, Germany

## Introduction

Caspase-1 activates proIL-1β and proIL-18 and plays a fundamental role in innate immunity. This pro-inflammatory immune response is required for the initiation of pathogen clearance. Caspase-1 itself is activated in so-called “inflammasomes” which are assembled in response to distinct pathogen associated molecular patterns (PAMP) or danger associated molecular patterns (DAMP). Most studies analyzing the inflammasome/caspase-1 activity of patients *ex vivo* use PBMC-based assays. Beside monocytes and macrophages, caspase-1 is also activated in PMNs representing the major leukocyte subset in peripheral blood. Thus, analyzing purified PBMCs seems to be highly artificial by excluding caspase-1 activity in PMNs and ignoring cellular interactions. Furthermore, PBMC purification requires large amounts of blood, thereby rendering the assay impractical for the use in small children or neonates.

## Objective

To establish and validate a small-volume based human whole blood assay facilitating the measurement of inflammasome-related gene expression and caspase-1 activation upon stimulation of the NLRP3, NLRC4 or AIM2 inflammasome.

## Methods

We set up a whole-blood assay in 96-well format using 140μl blood per well. Following stimulation with LPS/ATP, Salmonella typhimurium or LPS/polydA:dT (activation of NLRP3, NLRC4 or AIM2) IL-1β was measured in the supernatant and used as surrogate of caspase-1 activation. The inflammasome-specificity was studied for each stimulus by inflammasome inhibition using high potassium buffer, methylsulfonylmethane or MCC950. Furthermore, gene-expression analysis following red cell lysis was performed.

## Results

Using our optimized whole-blood assay, we are able to measure IL-1β secretion as well as gene-expression of *IL-1β, NLRP3, NLRC4, AIM2, PYCARD*, and *CASP1* following differential activation the NLRP3, NLRC4 or AIM2 inflammasome. Priming of cells with ultra pure LPS directly induced IL-1β secretion. This constitutive caspase-1 activity is already known for monocytes and depends on NLRP3. Further increase in NLRP3 activity was achieved using additional ATP stimulation, but this effect was dependent on continuous agitation of the probes. Secretion of IL-1β after stimulation with *S. typhimurium* was shown to be dependent on NLRP3 and NLRC4. In the early phase*, S. typhimurium* primed the cells and IL-1β secretion was mainly dependent on NLRP3 activity. Later on, IL-1β secretion was less susceptible to inhibition of NLRP3 inflammasome activity. Transfection of polydA:dT using Lipofectamine 2000 led to activation of the AIM2 inflammasome mainly. Interestingly, Lipofectamine without polydA:dT led to exclusive activation of NLRP3.

## Conclusion

It is possible to analyze caspase-1 activity and inflammasome-related gene expression in whole-blood samples following distinct inflammasome activation. Based on our data we assume that there is a superposition of NLRP3 and NLRC4 or AIM2 inflammasome activities in human whole blood following stimulation with *S. typhimurium* or polydA:dT.

This study was supported by the German Research Foundation (DFG, KFO 249) and by a MeDDrive project (University of Technology, Medical Faculty) to SW.

